# Novel Expression Vectors Enabling Induction of Gene Expression by Small-Interfering RNAs and MicroRNAs

**DOI:** 10.1371/journal.pone.0115327

**Published:** 2014-12-16

**Authors:** Liraz Harel, Nir Gefen, Ofira Carmi, Pini Orbach, Paz Einat, Guy Abitbol

**Affiliations:** 1 Nanodoc Biotechnology, 7 Sapir Str., Nes Ziona 7403630, Israel; 2 Dr. Paz Einat Biotechnology Projects & Consulting, 45a Moshe Levi Str., Nes Ziona 74207, Israel; The University of Tokyo, Japan

## Abstract

Small-interfering RNAs and microRNAs are small ∼21–22 nucleotide long RNAs capable of posttranscriptional suppression of gene expression. The synthetic siRNAs are especially designed to target pre-specified genes and are common molecular biology tools. The miRNAs are endogenous regulators of gene expression found in a wide variety of eukaryotes. miRNAs are currently utilized for diagnostics applications. Therapeutically, various miRNA-antagonizing tools are being explored and miRNAs are also utilized for cell-specific inhibition of the expression of gene therapy vectors harboring target sites for specific miRNAs. Here we show, for the first time, that siRNAs and miRNAs can be harnessed to induce gene expression. We designed special expression vectors in which target sites for artificial siRNAs or endogenous miRNAs are located between the transgene and an Upstream Inhibitory Region (UIR). We hypothesized that cleavage of the mRNA by siRNAs or miRNAs will separate the transgene from the UIR and the resulting uncapped mRNA will be capable of being translated. A UIR composed of seven open reading frames was found to be the most efficient inhibitor of the translation of the downstream transgene. We show that under such a configuration both artificial siRNAs and endogenous miRNAs were capable of inducing transgene expression. We show that using the diphtheria toxin A-chain gene, in combination with target sites for highly expressed miRNAs, specific induction of cell-death can be achieved, setting the stage for application to cancer therapy.

## Introduction

The discovery of RNA interference [1] led to the uncovering of a basic cellular regulatory mechanism common to a wide spectrum of organisms. Several families of small RNA were discovered including small interfering RNAs (siRNAs), microRNAs (miRNAs) and PIWI-interacting RNAs (piRNAs) [2].

RNA interference by long double-stranded (ds) RNAs turned out to be driven by small ∼21 nucleotides long RNA molecules derived from the long dsRNA [3], [4]. These small interfering RNAs (siRNAs) were shown to function also in mammalian cells as potent and specific inhibitors of gene expression and quickly became an important tool both experimentally and therapeutically.

MicroRNAs (miRNAs) were first discovered in 1993 [5] but their wide evolutionary abundance and regulatory roles were established at the beginning of our century [6]. These are ∼21–22 nucleotide long RNA molecules, derived from hairpin structure embedded in long mRNAs or introns of various genes. The miRNAs emerged as key regulators of a wide variety of biological processes. They function by binding to partially complementary sequences in mRNA transcripts, mostly in the 3′UTR, followed by inhibition of protein translation or exonucleolytic mRNA degradation. The vast majority of miRNA binding sites are characterized by complementarity to the “seed” region of the miRNA, corresponding to positions 2–8 with few additional pairing to other positions [7]. However, perfect or near perfect pairing of the miRNA to the mRNA target site leads to Argonaute-catalysed mRNA cleavage [8]–[10].

Expression profiling identified varied expression of miRNAs in normal tissues and deregulation of specific miRNAs in disease [11]–[13]. These studies established miRNAs as anchors for the development of diagnostic tools and as targets for the development of therapeutics. miRNAs were also identified in some viruses of which the herpesvirus family is notable for expressing multiple miRNAs [14]. While the exact role of viral miRNAs is still to be fully deciphered their existence in tumor-associated viruses such as Epstein-Barr virus (EBV) [15]–[17], might make them interesting therapeutic targets.

There are a variety of applications that can benefit from tight cell-type specific transgene expression. These include expression of therapeutic genes in specific cell-types, such as hepatocytes, to eliminate immune response due to expression in other cell-types [18], expression of genes which can be toxic in non-target cells, and expression of toxins in efforts to specifically eliminate cancer cells [19]. So far, most studies used specific promoters to achieve cell-specific transgene expression. However, in most cases such promoters do not show the same tight cell-specificity they poses endogenously, possibly since they are not found in their native chromosomal environment or they lack some of the regulatory elements. The use of 3′UTR embedded miRNA target sites for tissue-specific miRNAs to decrease transgene expression in undesired cells [20], [21] is a novel approach but its applicability is limited by availability of tissue-specific miRNAs. Additionally, it is reasonable to assume that this system will not be able to decrease the expression of potent toxin genes below biological activity levels.

We searched for novel ways to achieve cell-specific induction of gene expression. Our aim was to induce gene expression based on the presence of specific nucleic acid molecules in the cells. For this purpose miRNAs seem ideal as they poses the potential, in conjunction with RISC, to cleave mRNAs that contain perfect or near perfect matching sequences [22]. We designed expression vectors with a unique architecture encoding a mRNA in which the transgene is found downstream to a translation inhibition region. We placed target sequences fully matching siRNAs or miRNAs between the transgene and the translation inhibition region and hypothesized that cleavage of the mRNA by siRNAs or miRNAs will relieve the transgene from the translation inhibition, enabling transgene translation. We provide proof that this unique construct architecture leads to induction of gene expression by both synthetic siRNAs and endogenous miRNAs in a wide variety of human cell-lines. Furthermore, we harnessed the miRNA expression pattern to induce miRNA-dependent cell death in multiple cancerous cell lines.

## Results

### a) Designing a construct with minimal transgene basal activity

**Figure 1:**
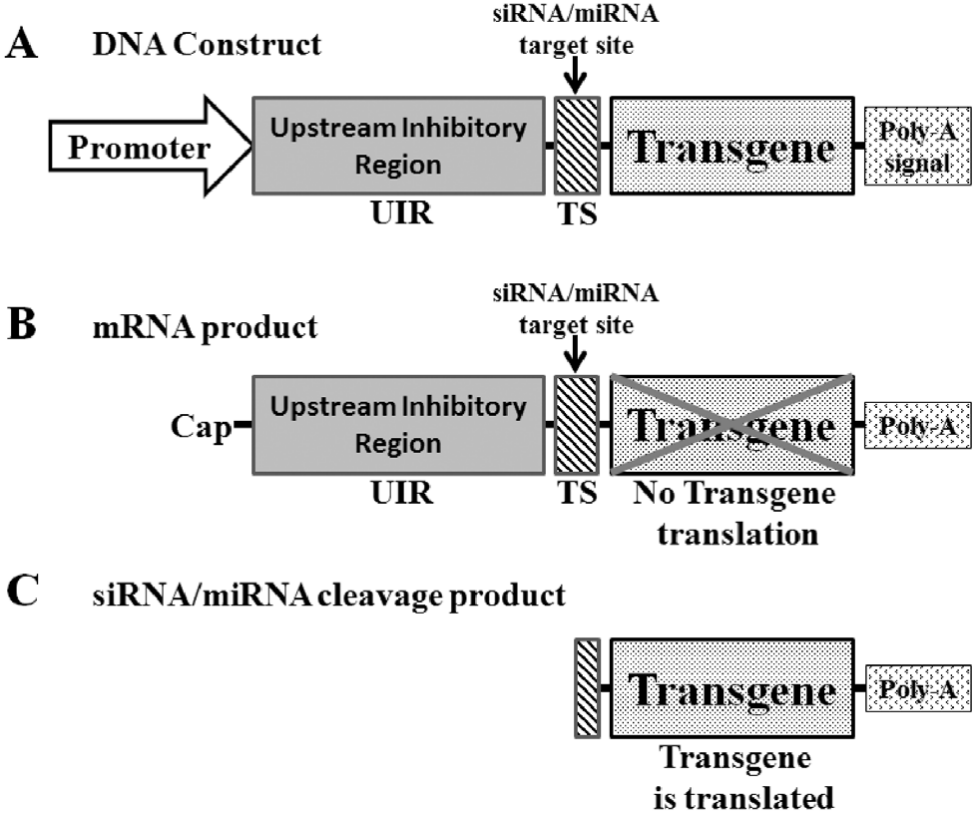
A scheme of the construct design aimed at siRNA or miRNA induction of transgene expression. (A) The design on the DNA level. (B) The construct on the mRNA level following transcription, no transgene expression due to the inhibitory effect of the UIR. (C) The mRNA product following cleavage by siRNA or miRNA. The cleaved product that includes the transgene contains half of the target site (TS), the UIR is cleaved away, and the transgene can be translated.

Our strategy to achieve transgene induction by siRNA and miRNAs involved placing a translation inhibition region (termed “Upstream Inhibitory Region” or UIR) upstream to the transgene and including a target site (TS) for siRNA or miRNA between the UIR and the transgene (Fig. 1A). In our design the UIR is composed of one or more upstream open-reading-frames (uORFs). We hypothesized that with such construct architecture the ribosomes will efficiently translate the uORFs, with minimal, if any, translation of the downstream transgene (Fig. 1B) [23]. Existence of siRNAs or miRNAs capable of cleaving the target site will split the mRNA, relieving the transgene coding region from the inhibitory effect of the UIR, and enable transgene translation (Fig. 1C). Indeed, the cleaved mRNA will lack a cap on its 5′ end, but such mRNAs are capable of being translated albeit at lower efficiency [24].

A prerequisite for achieving tight cell-type specific expression is as low as possible basal translation of the transgene’s ORF. We also reasoned that for achieving induction of gene expression by siRNA or miRNA we would need a large number of mRNA molecules of the transgene. Thus, we chose the strong CMV promoter/enhancer to drive the expression of the construct and we tested various compositions of the UIR for their inhibition of transgene translation. Our constructs included also a GFP gene cassette, intended initially as an internal control.

We tested several constructs in which the UIR was composed of 1 to 4 ORFs in the first frame and additional ORFs in the second frame (Table 1). The transgene’s ORF, always the most downstream one, was in the third frame. Each of the uORFs which composed the UIR was ∼600 bp long and contained 9 ATG codons in the same translational frame.

**Table 1:** The effect of various UIR configurations on the activity of the DTA transgene.

	UIR ORF’s		HUH7		HEK293T	
Construct	1^st^ Frame	2^nd^ Frame	REN l.u.	DTA activity	REN l.u.	DTA activity
No DTA			34,852,933		316,264,000	
ConstDTA	0	0	98,565	390	738,142	428
1ORF DTA	1	1	761,187	60	1,550,750	204
2ORF DTA	2	2	1,065,255	33	2,247,327	141
3ORF DTA	3	3	4,297,892	9	8,559,017	37
4ORF DTA	4	3	5,977,395	6	10,056,867	31

All construct had the CMV promoter before the UIR. REN l.u. = Renilla luciferase light units as determined by a luminometer. DTA activity is determined by dividing the REN l.u value of the “NO DTA” construct with that of each of the other constructs.

The transgene in our constructs encoded the A-chain of diphtheria toxin (DTA) [25], [26]. This toxin has a strong ADP-ribosyltransferase activity which catalyzes the transfer of NAD^+^ to a diphthamide residue in eukaryotic elongation factor-2 (eEF2), inactivating this protein. Thus, DTA is a highly efficient inhibitor of protein translation. Our assay involved co-transfection of the test plasmid with a reporter plasmid carrying the renilla and firefly luciferase genes (see M&M). DTA activity was measured by examining the inhibition of the renilla luciferase activity in cell extracts.

As can be seen in Table 1, a construct lacking a UIR, in which the DTA ORF is fully active, caused ∼400 fold inhibition of luciferase activity in both HUH7 and HEK293T cells. Addition of two uORFs reduced this activity by 6.5 fold and >2 fold, respectively, in the two cell lines, leaving a high residual DTA activity. Inclusion of additional uORFs caused gradual decrease of DTA activity. The lowest activity of the DTA transgene was observed in the 4ORF construct which includes four ORFs in the first frame and 3 ORFs in the second frame. Addition of more ORFs to the UIR, thus having more than 7 uORFs, did not cause additional reduction in the basal activity (data not shown). In addition, we found that the inclusion of many ATG codons in each of the ORFs had an additive effect in suppressing transgene expression (data not shown).

Since expression of the DTA transgene inhibits its own translation, the difference between the maximal expression (ConstDTA) and minimal expression (4ORF DTA), shown in Table 1, may not reflect the real difference between the designs of these two constructs. Thus, we examined the same constructs using the renilla luciferase as the transgene. As shown in Table 2, the addition of the 4ORF region (with 7 uORFs) reduced luciferase activity by ∼17,000 fold.

**Table 2:** Suppression of the activity of the renilla luciferase transgene by the 4ORF UIR.

Construct	REN l.u.	Fold reduction
pCMV-4ORF	1,762	
pCMV-REN	356,689,514	
pCMV-4ORF-REN	20,999	16,986

REN l.u. = Renilla luciferase light units as determined by a luminometer. The fold reduction was determined by dividing the luciferase light units obtained from the pCMV-REN construct by that obtained from the pCMV-4ORF-REN construct.

### b) Analysis of transgene induction by siRNAs

We included in the UIR-containing constructs target sites for two different siRNAs between the UIR and the DTA transgene. This allowed the testing of our hypothesis that in such construct configuration, siRNAs might be able to induce transgene expression.

**Figure 2:**
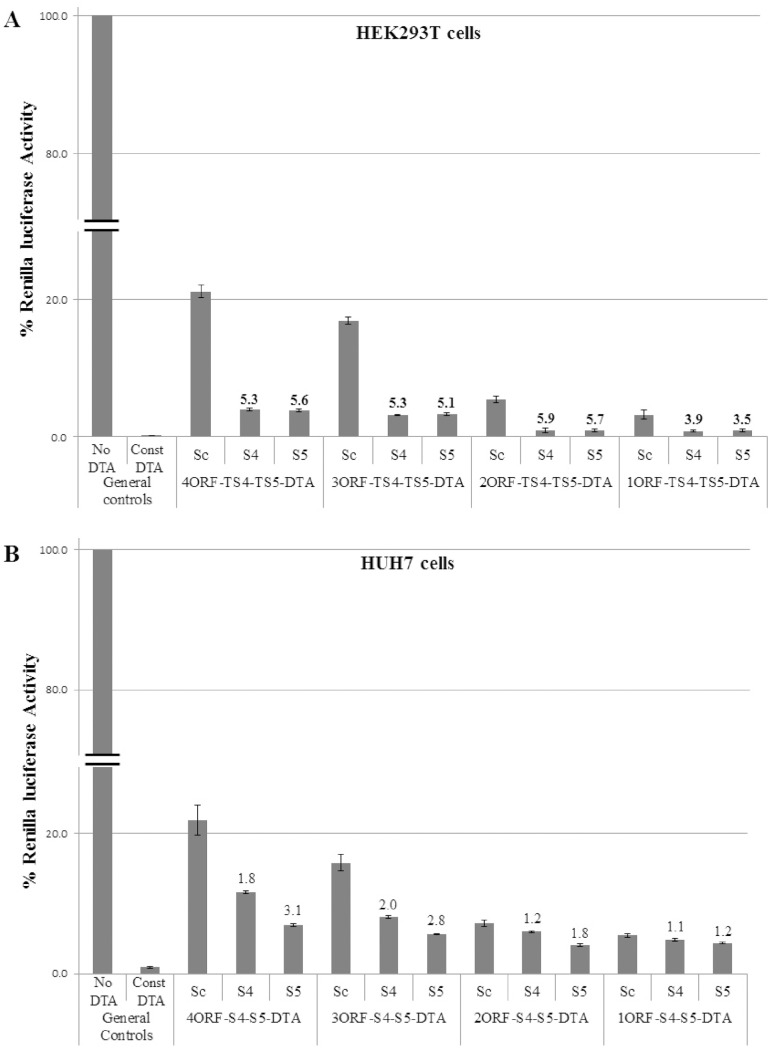
Transgene activation by siRNAs in constructs containing varied numbers of upstream ORFs. (A) Activity of the constructs in HEK293T cells. (B) Activity of the constructs in HUH7 cells. HEK293T cells (1.1×10^5^) were transfected with the indicated DTA construct (30 ng), a luciferase reporter plasmid (170 ng) and the indicated siRNA (10 pmol). HUH7 cells (8×10^4^) were transfected with the indicated DTA construct (50 ng), a luciferase reporter plasmid (150 ng) and the indicated siRNA (10 pmol). The renilla luciferase activity of the “No DTA” construct was set as 100% and the luciferase activity obtained for all other constructs were compared to the No DTA level. All constructs had the CMV promoter. The “No DTA” construct had the UIR but had no transgene. The “Const DTA” construct had no UIR, with the DTA transgene directly driven by the CMV promoter. The siRNAs used were: Sc: control siRNA; S4: siRNA S4; S5: siRNA S5. The numbers above the bars of the S4 and S5 siRNA indicated the DTA induction level calculated by dividing the luciferase activity of the Sc siRNA with that of the S4 or S5 siRNA of the same set. The experiments were repeated twice and each experiment was done in triplicates. Statistical analysis was done by T-test and the bars represent standard error. P values in A were 0.005; 2*10(−19); 2*10(−21) for 2ORF, 3ORF and 4ORF respectively. P values in B were 0.008; 6*10(−11); 6*10(−12) for 2ORF, 3ORF and 4ORF respectively. In both cases comparison was to the siRNA controls (Sc).

**Figure 3:**
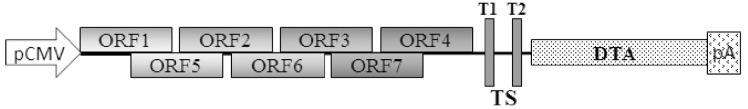
Structure of the selected UIR construct. Each of the 7 ORFs was ∼600 bp long. ORFs 1–4 were in frame 1, ORFS 5–7 were in frame 2, and the DTA transgene ORF was in the 3^rd^ frame. T1 and T2 are target sites (TS) for siRNAs. In constructs containing target sites for miRNAs there were usually 3 target sites. Full sequence and information on this construct, with target sites for siRNAs S4 and S5 can be found in the supplementary information).

HEK293T and HUH7 cells were transfected with expression vectors containing target sites TS4 and TS5 for siRNAs S4 and S5, respectively. Each transfection included the luciferase reporter vector and one of the siRNAs or a control siRNA. We compared the effect of the specific siRNAs S4 or S5 on DTA activity levels to that of control siRNAs (siCont) that had no target sites in the expression vector. As mentioned above, elevation of DTA transgene activity is manifested by inhibition of luciferase reporter activity. In HEK293T cells clear induction of DTA activity by the specific siRNAs, of 3.5 to 5.9 fold when compared to siCont, was observed in all four constructs (Fig. 2A). The 2ORF, 3ORF and 4ORF constructs had better induction levels than the 1ORF construct, with the 4ORF construct having the lowest DTA basal activity. In HUH7 cells we observed clear induction of transgene activity in the 4ORF-DTA and 3ORF-DTA constructs with both S4 and S5 siRNAs (Fig. 2B). Only marginal siRNA induction was observed with the 2ORF-DTA construct and no induction was seen with the 1ORF-DTA construct. These results suggest that significant transgene activation by siRNAs can be achieved in our construct configuration on the background of low basal transgene activity. The 4ORF construct (Fig. 3), which had similar induction levels as the 3ORF construct but lower basal activity level, was selected for the rest of our studies (see S1 File for detailed structure and sequence). Similar siRNA activation results with the 4ORF-DTA construct were obtained in many other cell-lines including HepG2 & PLC/PRF5 (human liver cancer), ES-2 (human ovarian cancer), T98G & U251 (human glioma), H1299 (human lung cancer), PC3 (prostate cancer), and HEK293 (data not shown).

**Figure 4:**
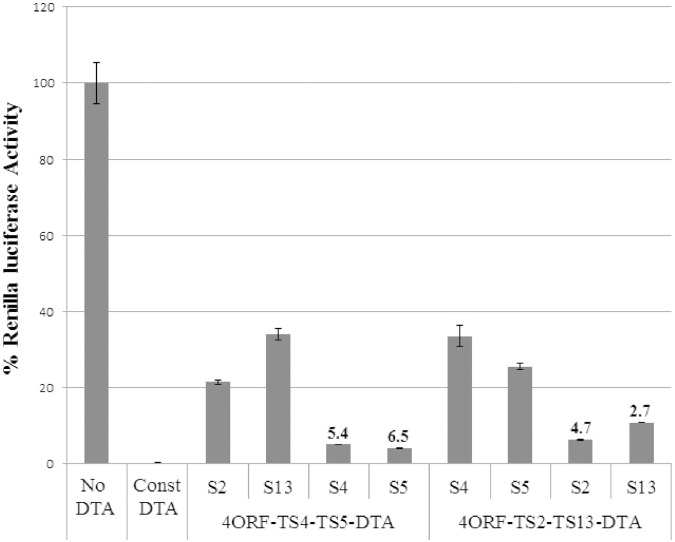
Reciprocal DTA activation by exogenous siRNAs in HEK293T cells. HEK293T cells (1.1×10^5^) were transfected with the indicated DTA construct (30 ng), a luciferase reporter plasmid (170 ng) and the indicated siRNA (10 pmol). The renilla luciferase activity of the “No DTA” construct was set as 100% and the luciferase activity obtained for all other constructs were compared to the No DTA level. The “Const DTA” construct had no UIR, with the DTA transgene directly driven by the CMV promoter. The siRNA used were: s4: siRNA S4; s5: siRNA S5; s2: siRNA s2; s13: siRNA S13. SiRNAs S2 and S13 served as controls for the 4ORF-TS4-TS5-DTA construct and siRNA S4 and S5 served as controls for the 4ORF-TS2-TS13-DTA construct. The numbers above the bars of the targeted siRNAs indicated the DTA induction level calculated by dividing the luciferase activity of the average of the control siRNAs with that of the indicted targeted siRNA of the same set. The experiment was repeated 3 times and each experiment was done in triplicates. Statistical analysis was done by T-test and the bars represent standard error. P values are <0.002 for S4 and S5 on the left side and S2 and S13 on the right side.

To obtain a conclusive proof for the ability of siRNAs to induce transgene expression in our construct configuration we designed additional 4ORF-TS-DTA constructs that included two target sites for different siRNAs. Thus, one construct had target sites TS4 and TS5 for siRNAs S4 and S5 and the other construct had target sites TS2 and TS13 for siRNAs S2 and S13. HEK-293 cells were transfected with the expression vector, the reporter vector and one of the siRNAs. As shown in Fig. 4, only siRNAs that had target sites in the vector caused an elevation in DTA translation inhibition activity and consequently were able to reduce renilla activity. Inclusion of siRNAs S4 or S5 in the transfections led to increased DTA activity in the 4ORF-TS4-TS5-DTA construct but not in the 4ORF-TS2-TS13-DTA construct and, conversely, inclusion of siRNAs S2 or S13 in the transfections led to induction of DTA activity of the 4ORF-TS2-TS13-DTA construct but not in the 4ORF-TS4-TS5-DTA construct. This reciprocal experiment provides a conclusive proof for the ability of siRNAs to induce transgene activity in our unique construct configuration. Thus, we can conclude that our unique construct architecture enables to achieve, for the first time to our knowledge, induction of gene expression by siRNAs.

**Figure 5:**
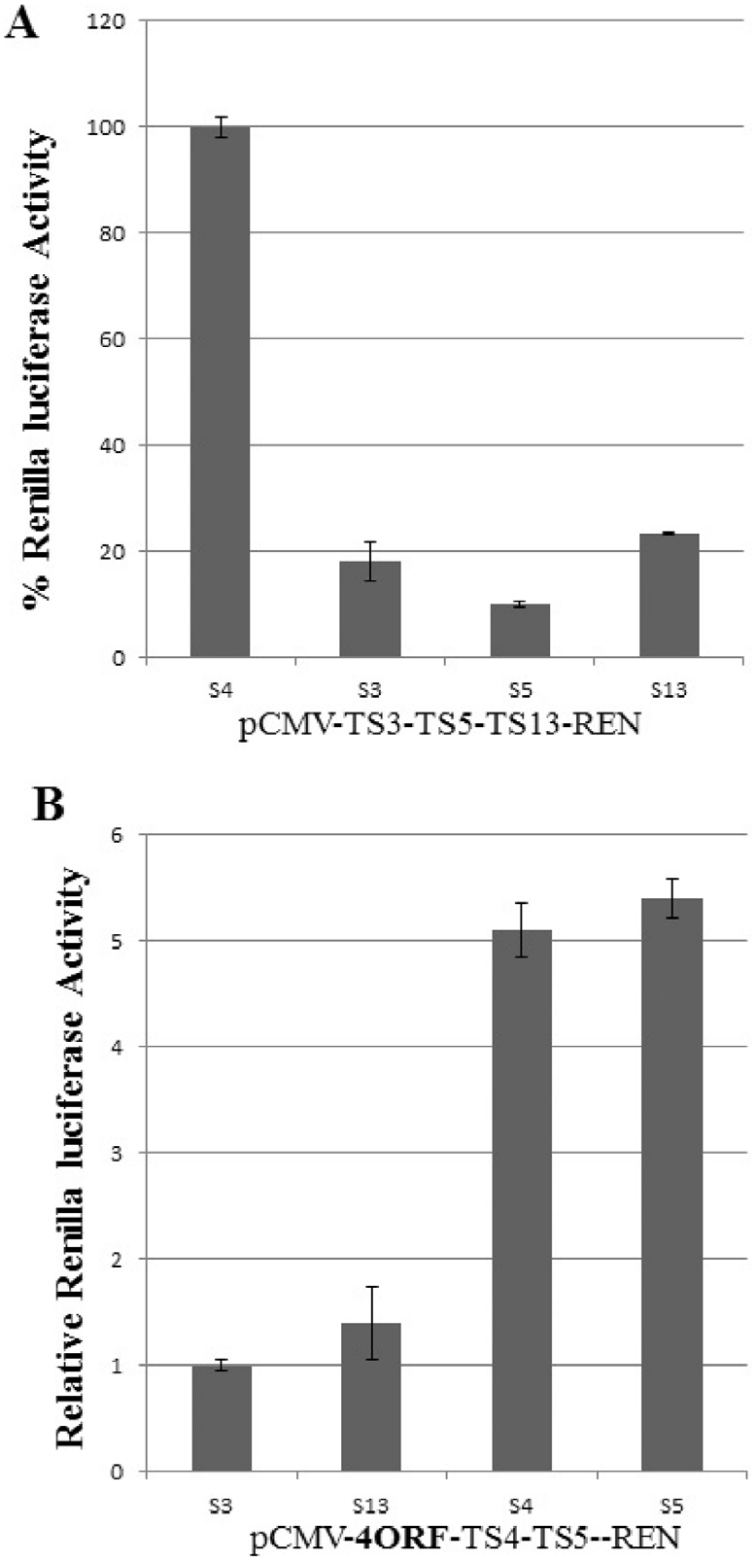
Induction of a renilla luciferase transgene by siRNA. (A) Three siRNA target sites were placed upstream the renilla luciferase reporter gene, without the UIR, and tested for the known standard siRNA activity. (B) Effects of siRNAs on renilla luciferase reporter gene in a construct containing the 4ORF UIR. In (A) the luciferase activity of the control S4 siRNA was set as 100%. In (B) the luciferase activity of the control S3 siRNA was set as 1 and the luciferase activity values obtained with each of the other siRNAs were compared to that. The experiments were repeated 3 times and each experiment was done in triplicates. Statistical analysis was done by T-test and the bars represent standard error. P Values in A are 2*10(−5); 1*10(−5); 0.002 for S3, S5 and S15, respectively. P Values in B are 0.0008; 1*10(−6) for S3 and S5, respectively.

In the experiments described above the induction by siRNAs was measured by following DTA translation inhibition activity through a luciferase reporter. To achieve a more direct measure of siRNA induction we replaced the DTA with the renilla luciferase reporter gene. Thus, activation with siRNAs should be reflected as an increase in luciferase activity. The siRNAs, for which target sites were included in the construct between the UIR and the transgene, were tested initially for standard siRNA activity in a construct lacking the UIR, the only difference being that the siRNA target sites were in the 5′UTR and not the 3′UTR. In this construct target sites for siRNAs S3, S5, and S13 were present. As shown in Fig. 5A, the three siRNAs reduced reporter activity by 4.3 to 7.4 fold as expected from active siRNA. In the UIR containing construct, in which target sites for siRNAs S4 and S5 where included, the two siRNAs caused a ∼4.5 fold increase in luciferase activity when compared to non-targeted siRNAs (Fig. 5B). Interestingly, such direct activation of reporter activity was observed only in HEK-293T cells. In a variety of other human cell-lines, including HEK-293, such direct induction was not observed although when DTA was used as a transgene most cell-lines demonstrated siRNA inductions.

### c) Induction of gene expression with endogenous microRNAs

The main aim of our approach was to achieve transgene induction by endogenous miRNAs. To examine whether our construct design is capable of supporting such induction, we placed specific miRNA target sequences between the UIR and the transgene. These sequences were fully complementary to miRNAs expressed in the cells used in our experiments. The cell-lines used were subjected to miRNA profiling (Table 3) and constructs containing target sequences matching miRNAs expressed at high levels and miRNA expressed at low levels were designed and constructed.

**Table 3:** MicroRNA expression profiling in various cancer cell-lines.

	Cell-line				
miRNA	U251	T98G	HepG2	HUH7	PLC/PRF5
hsa-miR-105-5p	38	20	6	8	123
hsa-miR-31-3p	191	267	0	2	1
hsa-miR-193a-3p	69	839	168	111	157
hsa-miR-10b-5p	1797	511	25	0	7
hsa-miR-10a-5p	231	100	0	0	1
hsa-miR-221-3p	11,601	7,003	461	1,371	3,614
hsa-miR-125b-5p	13,471	11,564	14	1,165	150
hsa-miR-1273g-3p	8,700	11,847	3,617	5	7,608
hsa-miR-21-5p	14,947	14,887	15,921	14,517	11,717
hsa-miR-9-5p	15,653	2,016	5	11	576
hsa-miR-15b-5p	4,358	4,027	1,748	2,078	1,815
hsa-miR-16-5p	ND	ND	2,657	3,005	2,784
hsa-miR-937	100	277	101	144	ND
hsa-miR-940	47	35	100	8	ND
hsa-miR-4752	1	5	ND	42	ND
hsa-miR-3613	1,521	1,627	10,584	15,515	9932
hsa-miR-4668	1,172	3,818	7,335	12,945	11874
hsa-miR-363	598	0	8,124	7	ND
hsa-miR-209	2,756	5,750	7,068	2,750	2634
hsa-miR-23a	28	40	2,819	8,089	8816
hsa-miR-192	84	74	2,958	2,376	7247
hsa-miR-23b	9	14	3,363	8,079	8105

MicroRNA expression profiling was done on the µParaflo Biochip of LC Sciences. The table present fluorescent values obtained for the listed miRNAs. Microarray data were verified by repetition in selected cell-lines and by quantitative RT-PCR (data not shown).

Initially, we included in each construct target sites for three different miRNAs. The liver cancer cell-lines HUH7 and HepG2 were transfected with the DTA constructs together with the luciferase reporter plasmid. Vectors containing target sites for siRNAs (see section “b”) were used as controls and luciferase activity levels of the vectors containing target sites for miRNAs were compared to the control. As described above, increase in the expression of the DTA transgene is manifested as decreased luciferase activity.

**Figure 6:**
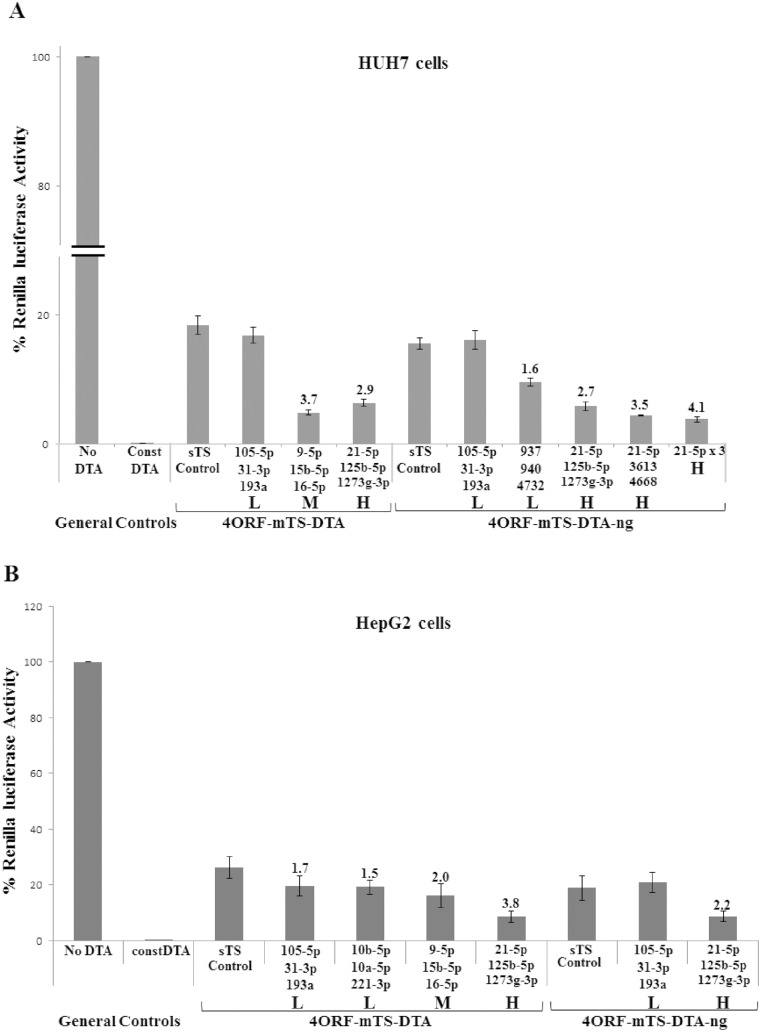
Induction of DTA by endogenous miRNAs. (A) Experiments in HUH7 cells. (B) Experiments in HepG2 cells. HUH7 cells (8×10^4^) were transfected with the indicated DTA construct (50 ng), a luciferase reporter plasmid (150 ng). HepG2 cells (8×10^4^) were transfected with the indicated DTA construct (175 ng), a luciferase reporter plasmid (25 ng). The renilla luciferase activity of the “No DTA” construct was set as 100% and the luciferase activity obtained for all other constructs were compared to the No DTA level. The “Const DTA” construct had no UIR, with the DTA transgene directly driven by the CMV promoter. Each bar on the X-axis stands for a construct containing different TS region. The “sTS” control was the 4ORF construct with target sites for siRNAs S4 and S5. The miRNAs for which fully matched target sites were included in the TS are indicated below the bars. The relative level of expression of the miRNAs in the cells (see Table 3) are marked as L (Low), M (Medium) or H (High). The numbers above the bars indicate the fold reduction of luciferase activity (and thus induction of DTA activity) between the relevant construct and the sTS control construct. The experiments were repeated 3 and 5 times, respectively, for A and B. Each experiment was done in triplicates. Statistical analysis was done by T-test and the bars represent standard error. P values in A were P<0.003 for all data points. P value in B were <0.01 for the two constructs containing the miRNAs 21-5p, 125b-5p, 1273g-5p target sites. For the construct containing target sites for miRNAs 9-5p, 15b-5p, 16-5p the p Value was 0.24.

Fig. 6A shows the results obtained in HUH7 cells. The construct containing target sites for miR-105-5p, miR-31-3p & miR-193a, all expressed at low levels in these cells, demonstrated DTA activity levels similar to those of the control construct. In contrast, the construct containing target sites for miR-21-5p, miR-125b-5p & miR-1273g-3p, all expressed at high levels, gave 2.9 fold higher DTA activity. Interestingly, in these cells also a construct with target sites for two miRNAs expressed at medium levels (miR-15b-5p & miR-16-5p) and one expressed al low level (miR-9-5p) gave higher DTA activity (Fig. 6A).

We examined additional constructs (4ORF-mTS-DTA-ng) from which we removed the GFP gene cassette in order to reduce plasmid size. In addition to the construct containing target sites for the lowly expressed miR-105-5p, miR-31-3p & miR-193a, which gave control level DTA activity, inclusion of target sites for the lowly expressed miR-937, miR-940 & miR-4732 also gave DTA activity similar to control levels (Fig. 6A). As above, inclusion of target sites for the highly expressed miR-21-5p, miR-125b-5p & miR-1273g-3p resulted in 2.7 fold higher DTA activity. Constructs including target sites for other highly expressed miRNAs also gave higher DTA activity. Thus, inclusion of target sites for miR-3613 and miR-4668, in addition to that of miR-21-5p also gave higher DTA activity. In these cells the highest activity was obtained with a construct containing three consecutive target sites for miR-21-5p (Fig. 6A).

Similar results were obtained in HepG2 cells (Fig. 6B). Two different combinations of lowly expressed miRNAs (miR-105-5p, miR-31-3p, miR-193a & miR-10b-5p, miR-10a-5p & miR-221-3p) gave marginal, and statistically insignificant, increase in DTA activity. A construct containing target sites for miR-9-5p, miR-15b-3p & miR-16-5p, two of which are expressed at medium levels, gave a 2 fold increase in DTA activity. Finally, the construct with target sites for the highly expressed miR-21-5p, miR-125b-5p & miR-1273g-3p gave 3.8 fold higher DTA activity.

Overall, we observed a strong correlation between the nature of the target sites in the constructs and DTA activity: constructs with targets for highly expressed miRNAs demonstrated significantly higher DTA activity levels than those containing targets sites for miRNAs expressed at low levels in the cells. This strongly suggests that the hypothesized mechanism of action is responsible for the elevated levels of expression in the specific constructs.

**Figure 7:**
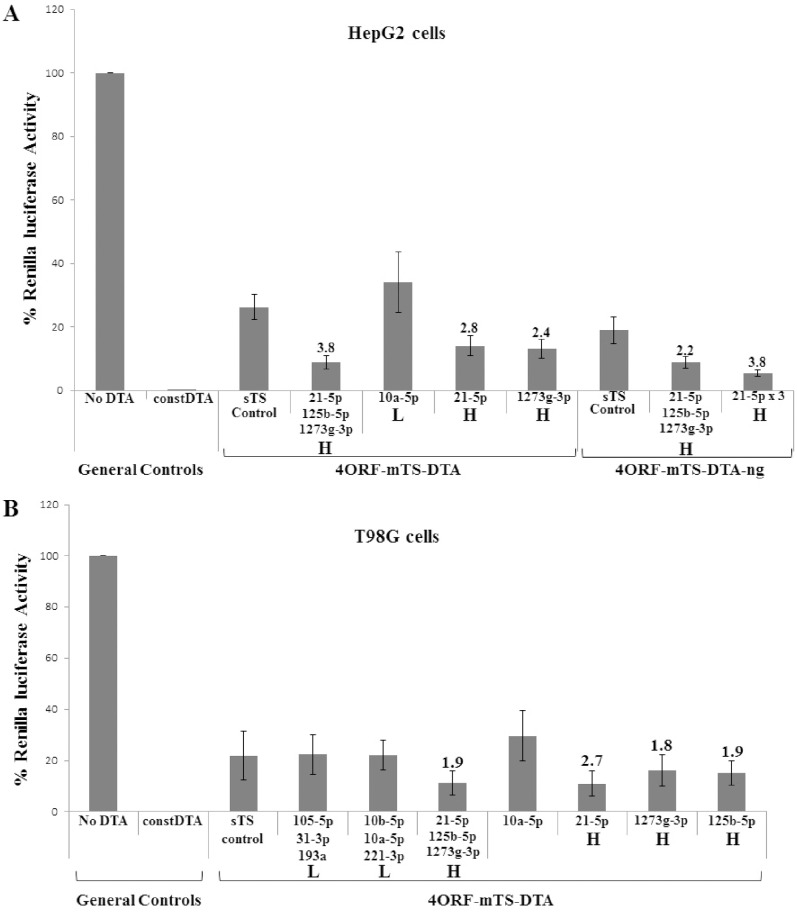
Induction of DTA by endogenous miRNAs in constructs containing single miRNA target sites. (A) Experiments in HepG2 cells. (B) Experiments in T98G cells. HepG2 cells (8×10^4^) were transfected with the indicated DTA construct (175 ng), a luciferase reporter plasmid (25 ng). T98G cells (5×10^4^) were transfected with the indicated DTA construct (50 ng), a luciferase reporter plasmid (150 ng). The renilla luciferase activity of the “No DTA” construct was set as 100% and the luciferase activity obtained for all other constructs were compared to the No DTA level. The “Const DTA” construct had no UIR, with the DTA transgene directly driven by the CMV promoter. Each bar on the X-axis stands for a construct containing different TS region. The “sTS” control was the 4ORF construct with target sites for siRNAs S4 and S5. The miRNAs for which fully matched target sites were included in the TS are indicated below the bars. The relative level of expression of the miRNAs in the cells (see Table 2) are marked as L (Low), M (Medium) or H (High). The numbers above the bars indicate the fold reduction of luciferase activity (and thus induction of DTA activity) between the relevant construct and the sTS control construct. The experiments described in A and B were repeated 3 times. Each experiment was done in triplicates. Statistical analysis was done by T-test and the bars represent standard error. P values in A for all construct with targets for highly expressed miRNAs (H) were <0.03. P values in B were significant for the construct with a target site for miR-21-5p (0.03) but not significant (>0.08) for the constructs with target sites for other miRNAs.

Most constructs described in Fig. 6 contain three target sites, each matching a different miRNA. We next tested whether targets for single miRNAs can support similar elevated DTA expression levels. We designed constructs that contained either one target site or three repeats of the same target site. As shown in Fig. 7, in both HUH7 and HepG2 cells single target sites for the same highly expressed miRNA (miR-21-5p & miR-1273g-3p) resulted in elevated expression levels of DTA. In comparison, DTA expression level in the construct containing target sites for both miRNAs (as well as for the lowly expressed miR-125b-5p) was higher than each of the single miRNA target constructs. As shown also in Fig. 6A, a construct containing triple miR-21-5p target sites had higher DTA activity than the single miRNA target vectors.

Similar results were observed in T98G glioma cells (Fig. 7B). Here, the single target vectors were at least as active as the vector containing target sites for the three highly expressed miRNAs, with the single miR-21-5p target site vector having the highest activity. No induction was observed for the vector containing a single target sites for the low-expressed miR-10a-5p. Thus, the same correlation of elevated DTA activity and presence of target sites for highly expressed miRNA exists also for the constructs containing a single miRNA target site.

### d) Induction of cell-death with constructs containing target sites for siRNAs or miRNAs

**Figure 8:**
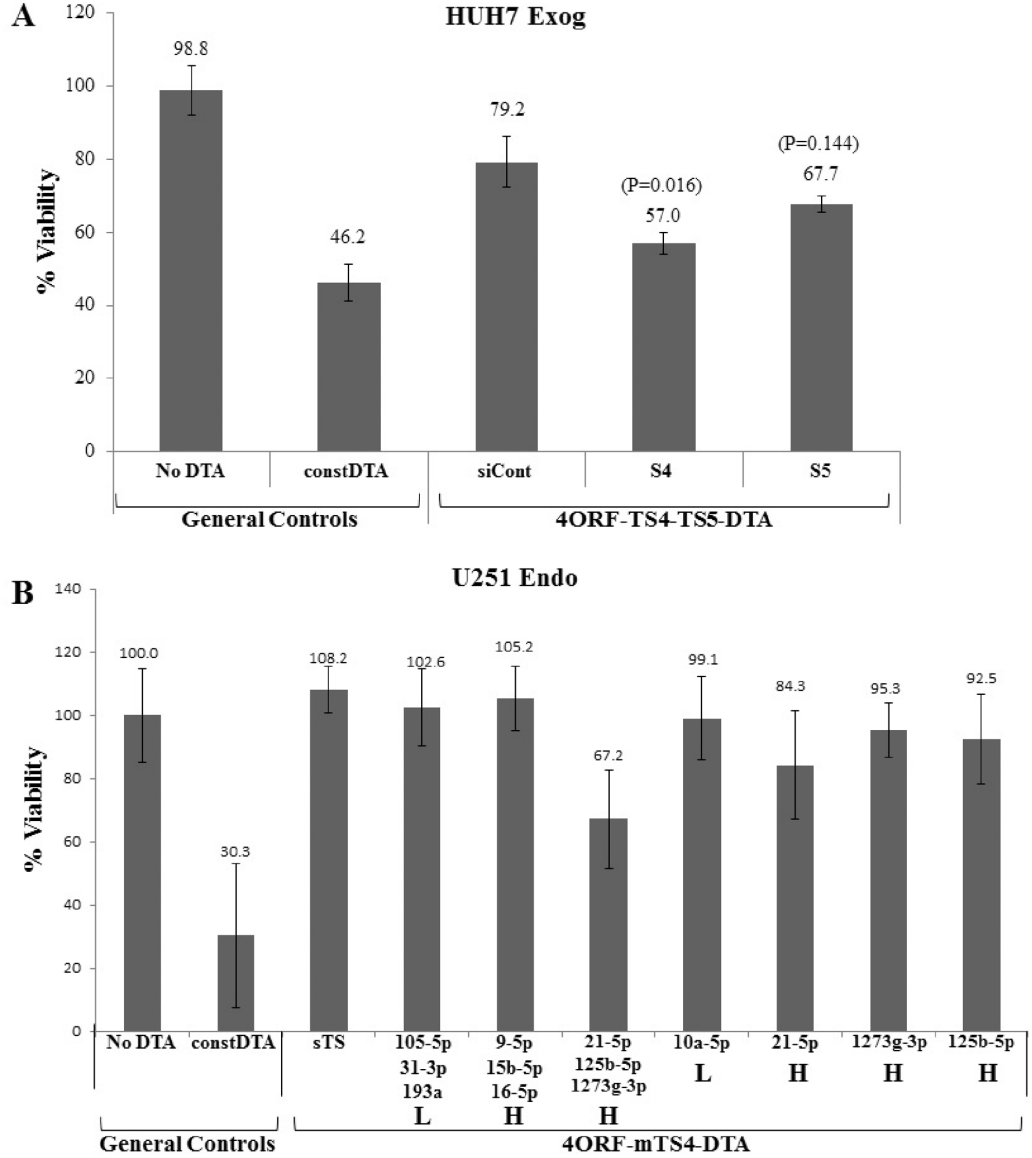
Decrease in cell viability by siRNA and miRNA dependent induction of DTA. U251 cells were transfected with the indicated construct (50 ng) with (A) or without (B) siRNAs (10pmol). Cell viability was determined by an XTT assay. The XTT viability value of the “No DTA” construct was set as 100% and the viability value obtained for all other constructs were compared to the No DTA level. (A) DTA dependent decrease in cell viability induced by siRNA in HUH7 cells. The siRNA used were: siCont: control siRNA; S4: siRNA S4; S5: siRNA S5. The numbers above the bars indicated the % viability obtained with each siRNA. P-values are indicated for the specific siRNAs. (B) DTA dependent decrease in cell viability induced by miRNA in U251 cells. The “Const DTA” construct had no UIR, with the DTA transgene directly driven by the CMV promoter. Each bar on the X-axis stands for a construct containing different TS region. The “sTS” control was the 4ORF construct with target sites for siRNAs S4 and S5. The miRNAs for which fully matched target sites were included in the TS are indicated below the bars. The relative level of expression of the miRNAs in the cells (see Table 2) are marked as L (Low) or H (High). The numbers above the bars of the siRNA indicated the % viability obtained with each siRNA. The experiments were repeated 3 and 4 times, respectively, for A and B. Each experiment was done in triplicates. Statistical analysis was done by T-test and the bars represent standard error. P value for the construct with target sites for miRNAs 21-5p, 125b-5p, 1273g-5p was 1.2*e(−10).

To examine whether the induction of DTA activity can lead to induction of cell death we measured the effects of our constructs, initially in combination with specific siRNAs, on HUH7 cell viability. Compared to reduction of ∼60% in cell viability by constitutively active DTA, the 4ORF-sTS-DTA construct with control siRNAs led to minimal decrease in cell viability (Fig. 8A). Addition of one of the specific siRNAs, S4, caused a significant decrease in cell viability. Addition of the second specific siRNA, S5, also caused a decrease in cell viability but this was of marginal statistical significance. We concluded that the basal activity of the 4ORF-TS-DTA had little effect on cell viability and that the specific siRNAs are able to induce DTA activity to a level which leads to significant cell-death.

We next examined the activity of our constructs in conjunction with target sites for endogenous miRNAs. We compared the effect on cell viability of constructs containing target sites for highly expressed miRNAs to that of constructs containing target sites for low-expressed miRNAs. The construct with targets sites for siRNAs (Fig. 8A) served as control. Fig. 8B shows the results obtained for U251 glioma cells. The vector expressing constitutively active DTA led to 70% decrease in cell viability. In comparison, the control vector, as well as the vector containing target sites for lowly expressed miRNAs (miR-105-5p, miR-31-3p, miR-193a, see Table 3) caused no decrease in viability levels. Thus, the basal DTA activity in these vectors had no effect on U251 cell viability. In contrast, presence of target sites for highly expressed miRNAs (miR-21-5p, miR-125b-5p & miR-1273g-3p; see Table 3) was associated with a significant decrease in cell viability of >30%. Modest effects on cell viability were observed for vectors that contained single target site for highly expressed miRNAs. The only exception was the construct containing target sites for miR-9-5p, miR-15b-5p and miR-16-5b, of which miR-9-5p is expressed at high levels (Table 3) and was not associated with decreased cell survival.

Decrease in cell viability by both siRNAs and miRNAs, in the respective constructs, was observed in most cell-lines mentioned above. Thus, our results strongly suggest that our unique construct architecture can be used for induction of cell-death of cells expressing high levels of the selected miRNAs.

## Discussion

Here we describe a unique construct architecture that enables, for the first time to our knowledge, the harnessing of siRNAs and miRNAs for induction of transgene expression. The unique construct architecture involves target sites fully matching specific siRNAs or miRNAs found between an Upstream Inhibitory Region (UIR) and a downstream transgene. We hypothesized that upon siRNA or miRNA-directed cleavage of the mRNA the transgene will be found on a cap-less RNA molecule capable of being translated. Since the translation efficiency of uncapped mRNAs is very low, and such RNAs are also less stable, there were two main requirements from the vector design. The first was that the selected transgene will have significant biological activity at low expression levels and the second was that there will be many mRNA molecules available. An additional prerequisite was a low basal activity, important for obtaining tight cell-specificity, and also, according to our hypothesis, for enabling transgene induction.

We used the diphtheria toxin A-chain (DTA) gene as a transgene. This toxin is a highly efficient inhibitor of protein translation and can lead to cell-death at very low expression levels [27]. This toxin is currently employed in various therapeutic strategies for cancer treatment including gene therapy approaches [19], [28], [29].

To achieve low basal transgene expression in our constructs we tested the effect of including various compositions of upstream open-reading-frames (uORFs) on the expression level of the downstream transgene. Previous studies have shown that short uORFs cause a significant decrease of transgene expression [23], [30]–[32]. In these and other studies short uORFs, often comprising only a few codons, were used and translation of the main ORF was reduced up to ∼20 fold. For our approach we required a much higher inhibitory level than previously reported. We tested UIRs composed of 2 to 7 uORFs, each ∼600 bp long, and found that the UIR containing the 7 uORFs had the highest inhibitory effect on transgene translation. Doubling this UIR to include 14 uORFs did not cause any further reduction in transgene translation. Thus, it is possible that there is a limit to the extent that transgene expression can be suppressed under such construct configuration. In addition, we found that inclusion of many ATG codons in each of the uORFs had an additive translation suppression value (data not shown). This UIR composition was the choice for our studies of siRNA and miRNA induction potential of our constructs.

Our construct with 7 uORFs, denoted 4ORF since it has 4 ORFs in the first frame, reduced transgene expression ∼400 fold in HEK293T and HUH7 cells when tested with the DTA transgene. This reduction in expression varied between various other cell lines and the maximal reduction level was ∼2,000 fold. As suggested in the studies using short uORFs, re-initiation of translation is a possible explanation for the basal low level of expression of the transgene in our constructs. Since the distance of the transgene’s ORF from the uORFs in our constructs is relatively high, it is also possible that low level of transcription initiation, from sequences just upstream of the transgene’s ORF, are also responsible for the basal expression.

When the basal level of expression of the 4ORF construct was examined in HEK293T cells with the renilla luciferase transgene we observed a reduction of ∼17,000 fold as compared to only ∼400 fold when DTA was the transgene. The reason for this difference is that DTA represses protein translation, including its own translation. Thus, in the construct lacking a UIR, DTA expression limits its own expression. This does not happen with the luciferase transgene. Thus, the difference in expression level between the construct lacking the UIR and the construct containing a UIR is significantly larger for the luciferase transgene and better reflects the overall suppression effect caused by the presence of the UIR.

Once we achieved a low basal activity construct, we could reliably test our construct for induction by siRNA or miRNA. We have shown that siRNAs can induce DTA expression in a variety of cell-lines. Of the four different siRNAs examined, only when the specific target sequence, fully matching the siRNA, existed in the construct did the siRNA cause induction of DTA activity. This provides a clear proof that siRNAs can induce gene expression in our unique construct architecture. The variability in the efficiency in which different siRNAs could induce DTA expression is expected. Such variability is a common feature of the siRNA field which uses them for gene repression and since the mechanism of action is the same the variability should also be similar.

While siRNA-dependent induction of the DTA transgene was observed in many human cell-lines, when we used the renilla luciferase as a transgene we observed inductions only in HEK-293T cells. Even in the parental HEK293 cells, lacking the SV40 T-antigen, no renilla luciferase induction could be observed. This is possibly related to the observation that DTA induction in HEK293T cells was consistently up to 2 fold higher than in other cell lines (Fig. 2A and Fig. 4). We can speculate that the difference between the DTA and luciferase transgenes in cell lines like HEK293 and HUH7 has to do with the fact that DTA suppresses its own translation. According to our main hypothesis, the additive expression from the uncapped mRNA produced by siRNA-directed cleavage can be observed only on a background of low basal expression from the uncleaved capped mRNA. This is due to the fact that uncapped mRNAs are translated at much lower efficiency then capped mRNAs. Thus, it is possible that the translation inhibition by DTA produces a further low basal activity, lower than that observed with the luciferase transgene, above which the additive translation of the cleaved mRNA is more pronounced. Why is this different in HEK293T cells? These cells express SV40 T-antigen which has direct effects on constructs containing the SV40 origin of replication. Since our constructs include this element it is highly likely that they are replicated as episomes, present in increased concentration in the transfected cells and, thus, expressed at higher level. SV40 T-antigen also has known effects on central cellular proteins controlling cell proliferation, including p53 and pRb proteins [33], [34]. It is possible that the effects on the cell cycle increases the rates of cap-independent translation, which has an important role during mitosis [35], are related to our observation. Other functions of SV40 T-antigen, such as the general effect on promoter activity [36], might also be relevant.

Our main aim was to achieve transgene induction by endogenous miRNAs. We observed a tight correlation between the identity of the miRNA target sites in the vector and the activity of the DTA transgene. Inclusion of target sites for miRNAs expressed at low levels result in DTA activity similar to that of control vectors. Importantly, constructs containing target sites for highly expressed miRNAs exhibited DTA activity levels 2–4 fold higher than that of the control vectors. We obtained such results in many cell-lines including HUH7 & HepG2 (liver cancer) H1299 (lung cancer), T98G & U251 (glioma), PC3 (prostate cancer), ES2 (ovarian cancer) and HEK-293. Since in these experiments we compared different vectors, each carrying a different set of miRNA target sites, it is difficult to obtain a conclusive proof that the observed inductions result from miRNA cleavage of the transgene mRNA. However, taken together with the conclusive results obtained with siRNA induction experiments, it is highly likely that the hypothesized mechanism of action was indeed responsible for the observed transgene inductions.

We compared the activity of our constructs with composite target sites for combinations of three different miRNAs, single target sites for specific miRNAs and three target sites for the same miRNA. While we did not cover all possibilities, our overall impression is that target sites for three different highly expressed miRNAs had some advantage over single target sites. Inclusion of three target sites for miR-21-5p, the highest expressed miRNA in most cell lines and a well-known oncomiR [37], gave DTA activity levels at least as high as the composite target sites. As expected, these findings varied from one cell-line to the other, possibly not only because of variation in miRNA expression levels. Clearly, more studies are required to determine the preferred composition of the target sites, but it is possible that each cell-line will have its unique preference.

The choice of DTA as a transgene was aimed at the outset to enable miRNA-specific cell death. We have shown that siRNAs are able to significantly induce DTA cell death in HUH7 cells. However, in these cells the basal activity of the construct had some effect on cell death. In the constructs designed for response to endogenous miRNAs we observed specific reduction of cell viability only when target sites for highly expressed miRNAs were included. In this case, using U251 glioma cells, the basal activity of DTA was not involved with detectable reduction in cell viability.

These results open up the possible application of our construct system for the development of cancer therapeutics. Inclusion of target sites for well documented cancer-enriched miRNAs such as miR-21 and miR-125b [37] is a clear possibility for various cancers but their low level expression in normal cells must be considered. Cancers with a strong viral involvement, such as the EBV-related gastric carcinoma [38], nasopharyngeal carcinoma [15] and Burkit’s lymphoms [17] are highly relevant therapeutic targets. In these cancers, targeting the EBV-derived miRNAs can provide highly specific targets that distinguish between normal and cancer cells. As mentioned above, even though a miRNA-dependent induction level of only ∼3 fold was observed for highly expressed miRNAs, it was enough to elicit an apparent specific effect on cell death. Further developments of our system may increase the induction level and enable not only a tighter cancer cell specificity but also application to other therapeutic areas.

## Materials and Methods

### Cell culture

The human cell lines HEK293, HEK293T, the human glioblastoma T98G, the human lung cancer H1299 and human ovarian cancer ES-2 were obtained from the American Type Culture Collection (ATCC; Rockville, MD). The human HCC cell lines, HepG2, PLC/PRF/5 and HUH7 were purchased from the JCRB cell bank (National Institute of biomedical Innovation) (Osaka Japan). The glioblastoma cell line U251 was purchased from the NCI Repository.

### siRNAs

All siRNAs were purchased from Dharmacon.

S2 (Cat.# P-002070-01-20) 5′-AAACAUGCAGAAAAUGCUGdTdT;

S3 (Cat.# D-002000-01-20) 5′-CUACACAAAUCAGCGAUUUUU;

S4 (Cat.# D-001400-01-20) 5′-CUUACGCUGAGUACUUCGA dTdT;

S5 (Cat.# P-002048-01-20) 5′-GCAAGCUGACCCUGAAGUUCAU

S11 5′-UCGCUUACCGAUUCAGAAUdTdT

S13 5′-CGCCAAGAACCUCAUCAUCUU

### Plasmid construction

All designed plasmids were sent for synthesis and cloning at Biomatik Corp. (Ontatio, Canada). The synthesis product was cloned into the pCMV6-A-GFP vector (OriGene) and the sequence of all derived plasmids was verified by sequencing.

### Transfection & luciferase assay

HEK293, HEK293T, H1299 & T98G were transfected using Lipofectamine2000 (Invitrogen). The U251 cell line was transfected using DarmafectDuo (Thermo Scientific). HepG2, PLC/PRF/5 & ES-2 cells were transfected with JetPrime (PolyPlus). All transfection were carried out according to the manufacturer protocol. In short, cells were plated in 24-well plates and allowed to reach confluency of 60–80%. The next day cells were co-transfected with psiCHECK-2 vector (Promega) and the Nanodoc test plasmids with or without siRNA. 48 hrs post transfection cells were harvested and luciferase activity was measured using the Promega dual luciferase reporter assay kit (E1960, Promega), and results were expressed as percentage from the No DTA control vector for each experiment in which the DTA transgene was used.

### Viability assay

Cell viability was determined 72 hours post transfection using the cell proliferation kit (XTT; BI, Israel). Results were expressed as percentage from the No DTA control vector of each experiment.

### Microarray analysis

For analysis of miRNA expression by microarrays 5×10^6^ cells were pelleted and sent for analysis at LC SciencesUsing the µParaflo MicroRNA microarray. The assay started from 1 µg total RNA sample were 3′-extended with a poly(A) tail using poly(A) polymerase. An oligonucleotide tag was then ligated to the poly(A) tail for later fluorescent dye staining. Hybridization was performed overnight on a µParaflo microfluidic chip using a micro-circulation pump (Atactic Technologies). On the microfluidic chip, each detection probe consisted of a chemically modified nucleotide coding segment complementary to target microRNA (miRBase version 20, http://mirbase.org) and a spacer segment of polyethylene glycol to extend the coding segment away from the substrate. The detection probes were made by *in* *situ* synthesis using PGR (photogenerated reagent) chemistry. The hybridization melting temperatures were balanced by chemical modifications of the detection probes. Hybridization used 100 µL 6×SSPE buffer (0.90 M NaCl, 60 mM Na_2_HPO_4_, 6 mM EDTA, pH 6.8) containing 25% formamide at 34°C. After RNA hybridization, tag-conjugating Cy3 dye were circulated through the microfluidic chip for dye staining. Fluorescence images were collected using a laser scanner (GenePix 4000B, Molecular Device) and digitized using Array-Pro image analysis software (Media Cybernetics). Data were analyzed by first subtracting the background and then normalizing the signals using a LOWESS filter (Locally-weighted Regression). microRNA expression levels below a value of 1000 were regarded as low and values above 10,000 were regarded as high.

## Supporting Information

S1 File **Sequence and description of plasmid pCMV-4ORF-DTA.** The sequence of the entire plasmid is presented. The presented DNA strand is the sense-strand with the regards to the CMV-4ORF-DTA expression unit.
(PDF)
