# Supplementary material for: Novel Expression Vectors Enabling Induction of Gene Expression by Small-Interfering RNAs and MicroRNAs
Source: PLoS One. 2014 Dec 16;9(12):e115327. doi: 10.1371/journal.pone.0115327 (PMC4267845; doi:10.1371/journal.pone.0115327)
Supplement: S1 File — Sequence and description of plasmid pCMV-4ORF-DTA. The sequence of the entire plasmid is presented. The presented DNA strand is the sense-strand with the regards to the CMV-4ORF-DTA expression unit. (PDF) [file pone.0115327.s001.pdf]

Plasmid pCMV-4ORF-DTA

Features:
Amp resistance: 563 – 1423
ColE1 replication origin: 1568 – 2240
P-Ori-SV40: 2648 – 2678
pCMV: 3135 – 3656
UIR: 3754 – 6272
    ORF1: 3754 – 4358
    ORF2: 4402 – 5043
    ORF3: 5053 – 5697
    ORF4: 5707 – 6537
    ORF5: 4121 - 4711
    ORF6: 4727 – 5365
    ORF7: 5381 – 6019
TS5: 6207 – 6234
TS4: 6235 – 6262
DTA: 6285 – 6872
hGH poly-A signal region: 6951 – 7063

```
AAAATTCGCGTTAAATTTTTGTTAAATCAGCTCATTTTTTAACCAATAGGCCGAAATCGGCAAAATCCCTT
ATAAATCAAAAGAATAGACCGAGATAGGGTTGAGTGTTGTTCCAGTTTGGAACAAGAGTCCACTATTAAAG
AACGTGGACTCCAACGTCAAAGGGCGAAAAACCGTCTATCAGGGCGATGGCCCACTACGTGAACCATCACC
CTAATCAAGTTTTTTGGGGTCGAGGTGCCGTAAAGCACTAAATCGGAACCCTAAAGGGAGCCCCCGATTTA
GAGCTTGACGGGGAAAGCCGGCGAACGTGGCGAGAAAGGAAGGGAAGAAAGCGAAAGGAGCGGGCGCTAGG
GCGCTGGCAAGTGTAGCGGTCACGCTGCGCGTAACCACCACACCCGCCGCGCTTAATGCGCCGCTACAGGG
CGCGTCAGGTGGCACTTTTCGGGGAAATGTGCGCGGAACCCCTATTTGTTTATTTTTCTAAATACATTCAA
ATATGTATCCGCTCATGAGACAATAACCCTGATAAATGCTTCAATAATATTGAAAAAGGAAGAGTATGAGT
ATTCAACATTTCCGTGTCGCCCTTATTCCCTTTTTTGCGGCATTTTGCCTTCCTGTTTTTGCTCACCCAGA
AACGCTGGTGAAAGTAAAAGATGCTGAAGATCAGTTGGGTGCACGAGTGGGTTACATCGAACTGGATCTCA
ACAGCGGTAAGATCCTTGAGAGTTTTCGCCCCGAAGAACGTTTTCCAATGATGAGCACTTTTAAAGTTCTG
CTATGTGGCGCGGTATTATCCCGTATTGACGCCGGGCAAGAGCAACTCGGTCGCCGCATACACTATTCTCA
GAATGACTTGGTTGAGTACTCACCAGTCACAGAAAAGCATCTTACGGATGGCATGACAGTAAGAGAATTAT
GCAGTGCTGCCATAACCATGAGTGATAACACTGCGGCCAACTTACTTCTGACAACGATCGGAGGACCGAAG
GAGCTAACCGCTTTTTTGCACAACATGGGGGATCATGTAACTCGCCTTGATCGTTGGGAACCGGAGCTGAA
TGAAGCCATACCAAACGACGAGCGTGACACCACGATGCCTGTAGCAATGGCAACAACGTTGCGCAAACTAT
TAACTGGCGAACTACTTACTCTAGCTTCCCGGCAACAATTAATAGACTGGATGGAGGCGGATAAAGTTGCA
GGACCACTTCTGCGCTCGGCCCTTCCGGCTGGCTGGTTTATTGCTGATAAATCTGGAGCCGGTGAGCGTGG
GTCTCGCGGTATCATTGCAGCACTGGGGCCAGATGGTAAGCCCTCCCGTATCGTAGTTATCTACACGACGG
GGAGTCAGGCAACTATGGATGAACGAAATAGACAGATCGCTGAGATAGGTGCCTCACTGATTAAGCATTGG
TAACTGTCAGACCAAGTTTACTCATATATACTTTAGATTGATTTAAAACTTCATTTTTAATTTAAAAGGAT
CTAGGTGAAGATCCTTTTTGATAATCTCATGACCAAAATCCCTTAACGTGAGTTTTCGTTCCACTGAGCGT
CAGACCCCGTAGAAAAGATCAAAGGATCTTCTTGAGATCCTTTTTTTCTGCGCGTAATCTGCTGCTTGCAA
ACAAAAAAACCACCGCTACCAGCGGTGGTTTGTTTGCCGGATCAAGAGCTACCAACTCTTTTTCCGAAGGT
AACTGGCTTCAGCAGAGCGCAGATACCAAATACTGTTCTTCTAGTGTAGCCGTAGTTAGGCCACCACTTCA
AGAACTCTGTAGCACCGCCTACATACCTCGCTCTGCTAATCCTGTTACCAGTGGCTGCTGCCAGTGGCGAT
AAGTCGTGTCTTACCGGGTTGGACTCAAGACGATAGTTACCGGATAAGGCGCAGCGGTCGGGCTGAACGGG
GGGTTCGTGCACACAGCCCAGCTTGGAGCGAACGACCTACACCGAACTGAGATACCTACAGCGTGAGCTAT
GAGAAAGCGCCACGCTTCCCGAAGGGAGAAAGGCGGACAGGTATCCGGTAAGCGGCAGGGTCGGAACAGGA
GAGCGCACGAGGGAGCTTCCAGGGGGAAACGCCTGGTATCTTTATAGTCCTGTCGGGTTTCGCCACCTCTG
```

ACTTGAGCGTCGATTTTTGTGATGCTCGTCAGGGGGGCGGAGCCTATGGAAAAACGCCAGCAACGCGGCCT
TTTTACGGTTCCTGGCCTTTTGCTGGCCTTTTGCTCACATGTTCTTTCCTGCGTTATCCCCTGATTCTGTG
GATAACCGTATTACCGCCTTTGAGTGAGCTGATACCGCTCGCCGCAGCCGAACGACCGAGCGCAGCGAGTC
AGTGAGCGAGGAAGCGGAAGAGCGCCCAATACGCAAACCGCCTCTCCCCGCGCGTTGGCCGATTCATTAAT
GCAGCTGGCACGACAGGTTTCCCGACTGGAAAGCGGGCAGTGAGCGCAACGCAATTAATGTGAGTTAGCTC
ACTCATTAGGCACCCCAGGCTTTACACTTTATGCTTCCGGCTCGTATGTTGTGTGGAATTGTGAGCGGATA
ACAATTTCACACAGGAAACAGCTATGACCATGATTACGCCAAGCTCTAGCTAGAGGTCGACGGTATACAGA
CATGATAAGATACAGCATGCTTTGCATACTTCTGCCTGCTGGGGAGCCTGGGGACTTTCCACACCCTAACT
GACACACATTCCACAGCCAAGCTGGCCGCGTACCCAATTCAACAGGCATCTACTGAGTGGACCCAACGCAT
GAGAGGACAGTGCCAAGCAAGCAACTCAAATGTCCCACCGGCTGGCGAAAGGGGGATGTGCTGCAAGGCGA
TTAAGTTGGGTAACGCCAGGGTTTTCCCAGTCACGACGTTGTAAAACGACGGCCAGTGCCAAGCTGATCTA
TACATTGAATCAATATTGGCAATTAGCCATATTAGTCATTGGTTATATAGCATAAATCAATATTGGCTATT
GGCCATTGCATACGTTGTATCTATATCATAATATGTACATTTATATTGGCTCATGTCCAATATGACCGCCA
TGTTGACATTGATTATTGACTAGTTATTAATAGTAATCAATTACGGGGTCATTAGTTCATAGCCCATATAT
GGAGTTCCGCGTTACATAACTTACGGTAAATGGCCCGCCTGGCTGACCGCCCAACGACCCCCGCCCATTGA
CGTCAATAATGACGTATGTTCCCATAGTAACGCCAATAGGGACTTTCCATTGACGTCAATGGGTGGAGTAT
TTACGGTAAACTGCCCACTTGGCAGTACATCAAGTGTATCATATGCCAAGTCCGCCCCCTATTGACGTCAA
TGACGGTAAATGGCCCGCCTGGCATTATGCCCAGTACATGACCTTACGGGACTTTCCTACTTGGCAGTACA
TCTACGTATTAGTCATCGCTATTACCATGGTGATGCGGTTTTGGCAGTACACCAATGGGCGTGGATAGCGG
TTTGACTCACGGGGATTTCCAAGTCTCCACCCCATTGACGTCAATGGGAGTTTGTTTTGGCACCAAAATCA
ACGGGACTTTCCAAAATGTCGTAATAACCCCGCCCCGTTGACGCAAATGGGCGGTAGGCGTGTACGGTGGG
AGGTCTATATAAGCAGAGCTCGTTTAGTGAACCGTCAGAATTTTGTAATACGACTCACTATAGGGCGGCCG
GGAATTCGTCGACTGGATCCATAGACGCGGTACCTAGCTAGGTAGCAATTGACGCGTCAAGATGGCGGCCA
ACAACAACAACAACAACAACAACAACAACAACAACAACAACAACAAGAAGATGGCGGCAACAACAACAACA
ACAACAACAACAACAACAACAACAACCAACAACAAGATGGCGGCCAACAACAACAACAACAACAACAACAA
CAAGAAGATGGCGGCAACAACAACAACAACAACAACAACAACCAAGATGGCGGCCAACAACAACAAGAAGA
TGGCGGCAACAACAACAACCAAGATGGCGGCCAACAACAACAAGAAGATGGCGGCAACAACAACAACCAAG
ATGGCGGCACGCTGCGGTCCGGCTAGCCGTACGCTCCTTAGCGACGAAATCTACTGTGAGATATCGAGCCA
CCATGGGATATCGTCCTACGCTGTGCAGGCCAAGTTTGGAGATTACAACAAAGAAGGCCGCCATGGTGGGC
ACCTCAGCTCTGAGCGGCTCATCCGCCACCATGGGTTGGACCAGCACAAACTTACCAGGGACCGCCGCCAT
GGCCGGACCCAGGCGTGCCACCATGGACACCGTGGGTTGCGCCGCCATGGTGCTCTGTTGGAGTGCCACCA
TGGTGCTCAGGACCTGGGCCGCCATGGAATACCTGATAACGTCCGGACCTGATAAGAGATCTTTGCCACCA
TGGGAACAGACCTTTGGCTTGGAGTTGACGCCCTTGGACTCAACATTTACGAGGCCGCCATGGAGTTCACC
CCAAAGATTGGCTTTCCTTGGAGTGAAATCAGGAACATCTCTGCCACCATGGAAAAGTTTGTCATCAAGCC
CATCGACAAGGCCGCCATGGACTTTGTGTTTTACGCCCCACGTTCCACAGCCACCATGGGGACCCTGCAGC
TCGCCGCCATGGACCACGAGTTGTACGCCACCATGGGGAAGCCTGACACCGCCGCCATGGAGCAGACGAAG
GCCGCCACCATGGCTGATAAGCTGATAACACGTCGCCGCCATGGGCCACGTCCAGAGAAGAAAAGGAGAGC
CGTGGAGAGAGAGAAAGAGCGCCACCATGGCGAGAAGGAGGAGTTGTTGCTGCGGCTGCAGGACTACGAGG
AGAAGACAAGCCGCCATGGGAGAGACCTCTCGGAGCAGATTCAGAGGGGCCACCATGGGGAGGAGGAGAGG
AAGCGGGCACAGGAGGGCCGCCATGGCCCAGAGGCTGACCGCCACCATGGACTGCGGGCTAAGGGCCGCCA
TGGGAGACAGGCGGTGGGCCACCATGGGAGCCAGGAGCAGCGCCGCCATGGTGATAACCCGTCTCTCCGGT
GATAAGCCACCATGGTGGAAGAGGCGCGGAGGCGCAAGGAGGACGAAGTTGAAGAGTGGCAGCAAGCCGCC
ATGGAAGCCCAGGACGACCTGGTCAAGACCAAGGAGGAGCTGCACCTGGTGCCGGCCACCATGGCGCCACC
ACCACCACCCGTGTACGAGCCGGCCGCCATGGACGTCCAGGAGAGCTTGCAAGACGAGGGTGCCACCATGG
CGGGCTACAGCGCAGCCGCCATGGCTGACGGCATCCGGGCCACCATGGACGAGGAGAAGCGTGCCGCCATG
GCAGAGAAGAACGAGGCCACCATGGGGCCTGATAAGCTGATAATCGCGAGCCGCCATGGGGTCGCGAACGA
GAACAAGAGGACCCACAACGACATCATCCACAACGAGAGCCACCATGGAGGCCGGGACAAGTACAAGACGC
TGCGGCAGATCCGGCAGGGCAACACCAGCCGCCATGGCGACGAGTTCGAGGCCCTGCAACAGCCAGGCCAC
CATGGAGGGCAGAGGGGTGCTCATAGCGGGCGCTGCCGCCATGGCCACGCTTGTGTCTGCCACCATGGAAG
TCTCGGAACTCGCCGCCATGGCAGTTCCTTTCGAAGCCACCATGGCAACAGAAACATTCGCCGCCATGGTG
ATAACCCCCGGGCCTGATAAGCCACCATGGTTGCAATCGTGCCAAGCAGGCCTGATTCTCGGCATTACTCG
CAGATCACCGCCGCCATGGTGCTGGGAGCAGGACTCATTGAATTACGGAAAACGCCTGTCAAGTCTCAGGC

CACCATGGGGAACTGGCCTGTGTCATACAAGAGTCAGGCCGCCATGGGGAAACGTGGCAGGACTTCCATCT
GTGCCGCCACCATGGTGTATTCGAAACGAGCCGCCATGGATTTTCTCATCTCTGCCACCATGGCATCTTTG
TACATTGCCGCCATGGGAGGGGTCAAAATTGCCACCATGGTGGCTGATAAGTTGATAGTAACCGCCATGGT
GTTTCATCCAGTCGCCACCATGGGCTGGCAGAGAGCAGCCGCCATGGCAGCGTCAGTGGTGGCCACCATGG
CTTGGATTTTTTTTTTTGTTTTTTTTTTTTTTGCTCAACAATTTTACAACACATTGTACCGGTCGAGCTCA
AGTCTCCGGCGCGCCCCGGTCCGTCCGGACTACGGCAAGCTGACCCTGAAGTTCATCCCAAAACTTACGCT
GAGTACTTCGATCTGGTCACCCCGGATCTCGCCGCCATGGGAGCTGATGATGTGGTTGATTCTTCGAAATC
TTTTGTCATGGAAAACTTTTCTTCGTACCACGGGACGAAACCTGGTTATGTGGATTCCATTCAAAAAGGCA
TACAAAAGCCAAAATCTGGTACACAAGGAAACTATGACGATGATTGGAAAGGGTTTTATAGTACCGACAAC
AAATATGACGCTGCGGGATACTCTGTGGATAATGAAAACCCGCTCTCTGGAAAAGCTGGAGGCGTGGTCAA
AGTGACGTATCCAGGACTGACGAAGGTTCTCGCACTAAAGGTGGATAATGCCGAAACTATTAAGAAAGAGT
TAGGTTTAAGTCTCACTGAACCGCTCATGGAGCAAGTCGGAACGGAAGAGTTTATCAAAAGATTCGGTGAT
GGTGCTTCGCGTGTAGTGCTCAGCCTTCCCTTCGCTGAGGGGAGTTCTAGCGTTGAGTACATCAACAACTG
GGAACAGGCGAAAGCGTTAAGCGTAGAACTTGAGATTAACTTTGAAACCCGTGGAAAACGTGGCCAAGATG
CGATGTATGAGTATATGGCTCAAGCCTGTGCAGGAAATCGTGTCAGGCGATAGTGAACTAGTATCCGGAAT
CTAGAGCGGCCGCACTCGAGGTTTAAACGGCCGGCCGCGGTCATAGCTGTTTCCTGAACAGATCCCGGGTG
GCATCCCTGTGACCCCTCCCCAGTGCCTCTCCTGGCCCTGGAAGTTGCCACTCCAGTGCCCACCAGCCTTG
TCCTAATAAAATTAAGTTGCATCATTTTGTCTGACTAGGTGTCCTTCTATAATATTATGGGGTGGAGGGGG
GTGGTATGGAGCAAGGGGCAAGTTGGGAAGACAACCTGTAGGGCCTGCGGGGTCTATTGGGAACCAAGCTG
GAGTGCAGTGGCACAATCTTGGCTCACTGCAATCTCCGCCTCCGTACATATTATGATATAGATACAACGTA
TGCAATGGCCAATAGCCAATATTGATTTATGCTATATAACCAATGACTAATATGGCTAATTGCCAATATTG
ATTCAATGTATAGATCAGCTTGGCACTGGCCGTCGTTTTACAACGTCGTGACTGGGAAAACCCTGGCGTTA
CCCAACTTAATCGCCTTGCAGCACATCCCCCTTTCGCCAGCTGGCGTAATAGCGAAGAGGCCCGCACCGAT
CGCCCTTCCCAACAGTTGCGCAGCCTGAATGGCGAATGGAAATTGTAAGCGTTAATATTTTGTT
